# Exogenous Substances Used to Relieve Plants from Drought Stress and Their Associated Underlying Mechanisms

**DOI:** 10.3390/ijms25179249

**Published:** 2024-08-26

**Authors:** Di Feng, Wenxin Liu, Ke Chen, Songrui Ning, Qian Gao, Jiao Chen, Jiao Liu, Xiaoan Sun, Wanli Xu

**Affiliations:** 1 Key Laboratory of Saline-Alkali Soil Improvement and Utilization (Saline-Alkali Land in Arid and Semi-Arid Regions), Ministry of Agriculture and Rural Affairs of the People’s Republic of China, Institute of Soil Fertilizer and Agricultural Water Conservation, Xinjiang Academy of Agricultural Sciences, Urumchi 830091, China; fengdi2008sunny@163.com (D.F.); 18811573974@163.com (J.C.); liuxy0803@163.com (J.L.); 2 School of Tropical Agriculture and Forestry, Hainan University, Haikou 570228, China; 15671779093@163.com; 3 College of Agriculture, South China Agricultural University, Guangzhou 510640, China; 13782901957@163.com; 4 State Key Laboratory of Eco-Hydraulics in Northwest Arid Region of China, Xi’an University of Technology, Xi’an 710048, China; ningsongrui@163.com; 5 Institute of Plant Protection, Chinese Academy of Agricultural Sciences, Beijing 100193, China; gao21318@163.com

**Keywords:** exogenous substances, drought tolerance, osmotic adjustment, antioxidant system, plant hormones

## Abstract

Drought stress (DS) is one of the abiotic stresses that plants encounter commonly in nature, which affects their life, reduces agricultural output, and prevents crops from growing in certain areas. To enhance plant tolerance against DS, abundant exogenous substances (ESs) have been attempted and proven to be effective in helping plants relieve DS. Understanding the effect of each ES on alleviation of plant DS and mechanisms involved in the DS relieving process has become a research focus and hotspot that has drawn much attention in the field of botany, agronomy, and ecology. With an extensive and comprehensive review and summary of hundred publications, this paper groups various ESs based on their individual effects on alleviating plant/crop DS with details of the underlying mechanisms involved in the DS-relieving process of: (1) synthesizing more osmotic adjustment substances; (2) improving antioxidant pathways; (3) promoting photosynthesis; (4) improving plant nutritional status; and (5) regulating phytohormones. Moreover, a detailed discussion and perspective are given in terms of how to meet the challenges imposed by erratic and severe droughts in the agrosystem through using promising and effective ESs in the right way and at the right time.

## 1. Introduction

With the abnormality of the global climate and the destruction of ecological balance, drought has become a key factor restricting agricultural development. Drought refers to the phenomenon of water shortage in the soil or atmosphere. As a common type of abiotic stress, drought causes billions of dollars in losses to global agriculture every year [1], which is more than that caused by low temperature and saline alkali stress combined together [2]. In addition, ecosystems suffer from drought stress (DS) due to loss of biodiversity, water depletion, soil desertification, and intensified climate changes [3]. The acreage of the arid region worldwide accounts for approximately 40% of the farm land now, but this percentage is rapidly growing with climate change [4]. Drought has affected an arid area of 2.976 × 10^6^ km^2^ in China, accounting for 38.3% of the total arable land, with an area of 6.97 × 10^5^ km^2^ in the extremely arid region [5]. DS has had a serious impact on agriculture by reducing crop yield/quality, making crops vulnerable to pests and natural disasters and devastating soil fertility through a reduction in microbial activity [6].

DS makes it difficult for plants to absorb sufficient water, hinders photosynthesis, slows down nutrient absorption and transport, and limits the cell elongation and metabolic activities [7]. When drought continues to be exacerbated, wilted root hairs further reduce water absorption and cause water loss in plants. Moreover, DS initially causes dehydration, stomatal closure, curling, and withering in leaves, thereby reducing photosynthesis efficiency and other leaf functions [8]. Subsequently, DS gradually interferes with physiological and metabolic processes such as chlorophyll production, protein synthesis, and energy metabolism. Abnormalities occurring in these physiological processes may lead to a hindered plant growth, weakened defensive responses, and accumulated harmful metabolites. To date, DS has been reported to cause damages to the turgor, membranes, and organelles of plant cells [9], thus resulting in a loss of cell integrity, causing cytoplasmic leakage, and distressing the normal growth and development of plants. DS can also disturb the balance of phyhormones, especially the ABA content, which is considered as a DS-responsive signaling molecule in plants. A properly increased ABA can boost plant tolerance against DS, but an excessive ABA accumulation may have an adverse effect on plant growth and development [10]. Moreover, exogenous genes extracted from other plants or modified through endogenous gene expression inside the plants have recently proven to improve plant resistance or tolerance against abiotic stresses. Among these genes used to alleviate plant DS, functional ones control important enzymes involved in detoxification and some metabolic proteins such as ion transporters and heat shock proteins, etc., while regulatory ones participate in the expression of various regulatory proteins (e.g., transcription factors, protein kinases, protein phosphatases) and signal transduction in response to DS [11]. In addition, plant defensive responses are correlated with microbial composition and activities within the rhizosphere [12]. The amount and nutritional profile of root exudates have proven to affect the dynamics of the microbial community, which in return boosts plant responses according to the onset of biotic or abiotic stresses. Therefore, plant tolerance against various stresses should be enhanced by changing the type of nutrients in or the nutritional profile of root exudates through adding beneficial microbes and encouraging their activities in the plant rhizosphere in soil under water deficit [13].

For the last half century, the exogenous substance (ES) has proven to be effective on relieving DS to some extent, and many fundamental mechanisms involved in DS alleviation through using ES have surfaced due to extensive and strenuous research by many scholars working in the area worldwide [14]. Some breakthroughs have revealed basic involvement of each ES in helping plants against DS based on understanding plant responses to drought [15]. The current research has mainly focused on understanding the effect of a single ES on certain individual crops to mitigate DS, but in the real world, a more comprehensive, systematic, and practical guideline is urgently needed for agricultural production under the circumstance of irregular drought occurrence through a review and summary of the most current publications during the last 24 years. With a description of 61 ESs reported in the literature and the details of their regulatory functions relating to plant responses against DS, this review article attempts to categorize the mechanisms of ESs involved in alleviating plant DS; analyze the effectiveness of various ESs based upon the information gathered from their application method, such as concentration, location, timing, plant species, and plant growth stage, etc., during drought; and provide relevant strategies for practically using ESs on plants under drought. We also share our future research perspectives.

## 2. Research and Development of Exogenous Substances to Alleviate Plant Drought Stress

### 2.1. Overview

**Figure 1:**
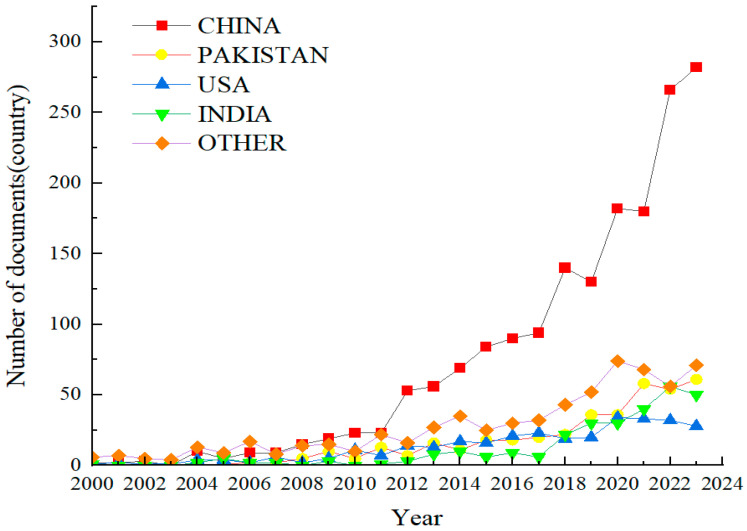
Published papers on drought stress in different countries from 2000 to 2023.

Exogenous substances include plant growth regulators, osmotic protectants, nutrients, and signaling molecules [16]. The research on ESs to alleviate plant DS began in the 1970s. With the keywords of both “drought stress” and “exogenous” to search relevant papers published from 2000 to 2023 in the Web of Science database, this paper found that a total of 3746 articles have been published and included in the core collection of Web of Science. From the perspective of inter-country comparison of the relevant papers published by different countries and institutions from 2000 to 2023 (Figure 1), Chinese, Pakistani, American, Indian, and other scholars from various countries have contributed 52.4%, 11.4%, 9.9%, 8.3%, and 15.7% papers, respectively. This trend of an increase in literature in different agricultural countries suggests that scholars have been paying more and more attention to the research on drought impacts and preventive strategies to meet the possible challenge imposed by DS on agriculture and the environment due to global climate change, agricultural water shortage, uneven distribution of annual precipitation, and intensified DS on plants.

### 2.2. Analysis of Research Hotspots and Frequently Used Exogenous Substances Worldwide

**Figure 2:**
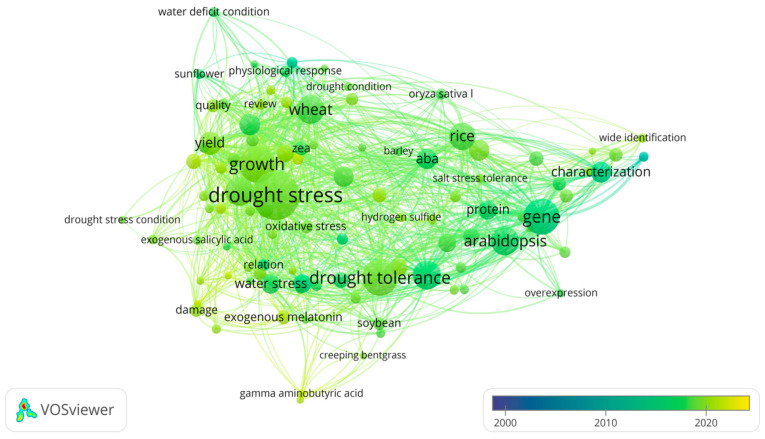
A hotspot analysis of research papers in English pertaining to the keywords of “drought stress” and “exogenous” from 2000 to 2023. Note: The size of each dot represents the focal weight of each keyword in the literature, and the lines between two dots indicate their coupling relationship.

A research hotspot analysis was performed using the VOSviewer(V1.6.18) software to visualize the historical and significant research focuses and their correlations based on all retrieved data (Figure 2). Four main research hotspots, “stress physiological”, “gene expression regulation plant”, “plant proteins”, and “abscisic acid”, stand out to be the most focused research fields, accounting for 606 (16.2%), 602 (16.1%), 476 (12.7%), and 420 (11.2%) papers, respectively. The analysis results indicate that in the past 24 years, research on the use of ESs to alleviate DS has focused on understanding the mechanisms involved in plant signal transduction, gene expression, protein conformation, and enzyme catalysis that regulate plant physiology and relevant metabolic pathways in responses to DS with the ES application. Based on this analysis, this review summarizes all ESs reported in recent literature; lists 61 commonly used and effective ESs for plant DS alleviation (Table 1); and divides them into three major categories: inorganic (multiple elements), organic (sugars, polyamines, plant growth regulators, phytohormones, signaling molecule, polyols, polyphenols, polypeptides, amino acids, and organic acids, etc.), and microorganisms. Among these ESs, ten have been shown to be the most frequently studied and discussed substances or subjects in research for the last 24 years, and abscisic acid (ABA) is on the top of the list, with various effects on mitigating plant DS (Table 2).

## 3. The Mechanisms of Exogenous Substances Involved in Alleviating Plant Drought Stress

Hundreds of ESs has been tried, and some of them have proven to participate in regulating plant structural, physiological, biochemical, and genetic responses against DS. With the outcome of the research hotspot analysis (Figure 2) and in reference to studies on the hormone-regulating mechanism and interaction of plant roots and their surrounding microbes that are believed to be involved in plant drought tolerance [17], the regulatory effects of ESs against plant DS seem to fall into 5 categories: inducing synthesis of osmotic regulators, regulating antioxidants, improving photosynthesis, and balancing phytohormones.

### 3.1. Induced Synthesis of Osmotic Regulators

Once under DS, plant cells generally lose water and accumulate osmotic adjusting substances, resulting in an increase in the cytoplasmic concentration and water retention capacity to reduce osmotic potential, maintain water absorption, and cell expansion against drought [18]. Two kinds of osmotic-regulating substances have been identified to be involved in the process of osmotic adjustment in plants. One is inorganic ions such as K^+^, Cl^−^, and other salts that rather freely enter plant cells from the external environment, and another group includes organic solutes synthesized inside cells such as proline, betaine, glycerol, etc., and some metabolic intermediates such as sugars and their derivatives [19].

Xu [20] found that an application of exogenous γ-PGA could significantly increase the proline concentration of rapeseed, maintain the stability of osmotic pressure, and thus maintain the water content in plants under DS. Similarly, exogenous addition of 100 mM NaCl promotes the Na^+^ accumulation in crops under DS to prime cell membranes for their stability through inducing accumulation of organic solutes such as proline, betaine, and soluble carbohydrates [21], while exogenous CTS more specifically promotes the accumulation of soluble sugar in leaves at the early stage of DS and prevents possible damage due to DS at the later periods [22]. K^+^ also participates in the sugar metabolic pathways of crops by increasing the cell osmotic concentration, maintaining the tension of stomatal guard cells, and promoting stomatal opening [23]. In addition, mineral nutrients such as selenium and silicon are of importance in plant metabolism and other physiological and biochemical processes (such as enzyme activity, osmotic regulators, protein synthesis, and photosynthesis) in plants. Therefore, reasonable supplementation of mineral elements can enhance the osmotic adjustment ability and antioxidant capacity of plants and achieve the effect of alleviating DS [24]. In terms of a symbiotic relationship between some plants and rhizosphere microorganisms, substances secreted by microorganisms prove to promote the osmotic regulatory mechanism in plant roots such as extracellular polysaccharides, which serve as a protective metabolite for plant cell membranes and enhance plant tolerance against abiotic stresses [25]. The commonly used ESs that induce the synthesis of osmotic regulators are shown in Table 3.

### 3.2. Improvement of Antioxidant Pathways

When subject to DS, plant cells gradually lose water and their mitochondria accumulate a large amount of ROS, which is detrimental to cell membranes composed of phospholipid bilayers, causing liquidation of the cell membranes and production of a large amount of MDA [7]. Both ROS and MDA distress the structure and function of organelles, distort biological macromolecules such as lipids and proteins, and breach the permeability of cell membranes [39], which activate endocrine antioxidant enzymes such as SOD, POD, CAT, etc., to protect plants from further serious damages due to excessive ROS and/or MDA [40]. In addition, plants can also alleviate oxidative damage induced by drought stress through increasing the non-enzymatic components of antioxidants, such as ascorbic acid, glutathione, carotenoids, tocopherols, flavonoids, and alkaloids, thereby enhancing the tolerance of oxidative stress induced by DS [41].

Many ESs have demonstrated that they can effectively mitigate the DS threat to plants under certain circumstances. The application of Pro proved to increase the photosynthetic rate of plant seedlings and balance the antioxidant metabolism [42], while Siddiqui [43] found that H_2_S had promoted the activity of antioxidant enzymes in leaves to reduce a potential loss of lipid peroxidation in plant cell membranes and improve the adaptability of plant seedlings against DS. Many antioxidant enzymes (SOD, POD, CAT) can be activated and promoted for more activities such as 10 μM SA [44] and 5-ALA [45] in different plant parts to remove extra ROS produced under DS. In addition, melatonin is widely recognized as a free radical scavenger and antioxidant [46,47], and its application significantly reduces the levels of H_2_O_2_, oxidation of lipid membranes, and accumulation of ROS and MDA [48]. Transcription factors are a group of regulatory components involved in gene expression of plant osmotic components and antioxidants to improve drought tolerance. Wang [49] divided the DS signal transduction into the ROS → MAPK and ROS → Ca^2+^ pathways. The former regulates the plant antioxidant and osmo-regulation system by clearing reactive oxygen species and cell osmotic potential, while the latter participates in the expression of plant-protective proteins such as LEA proteins through CDPK (calmodulin-dependent protein kinase) via the Ca^2+^ signaling channel [50]. An external application of the 0.5 mM salicylic acid significantly enhanced the transcription of *GST1*, *GST2*, glutathione reductase (*GR*), and monodehydroascorbate reductase (*MDHAR*) genes and improved plant drought resistance through modulating the ASA content and co-glutathione (GSH) cycle [51]. A putrescine treatment at a 0.3 mM concentration could regulate the gene expression coding for all SOD, CAT, and APX enzymes in both genotypes to improve plant tolerance against oxidative stress due to drought [52]. An excessive boron and water deficit can significantly stimulate the expression and signal transduction of the GR1, MT2, and *Hsp90* genes, significantly promoting APX and GR enzyme activity and enabling plants to initiate defensive responses earlier against various abiotic stresses [53]. When microorganisms in the soil sense the pressure caused by drought stress, they will secrete phenolic substances to induce the emergence of plant antioxidant systems to improve drought tolerance. An inoculation of two compatible plant-promoting rhizobacteria (PGPR), Pseudomonas putida (NBRIRA) and Bacillus valerate (NBRISN13), on plants under DS could induce the production of antioxidant enzymes, alleviate oxidative damage, and promote the abnormal accumulation of plant hormones [54]. All ESs that activate antioxidant enzymes and scavenge for removal of toxic ROS are listed (Table 4).

### 3.3. Promotion of Photosynthesis

The photosynthetic pigments, photosystem I and II, electron transport chain, and CO_2_ reduction pathway are four basic components involved in photosynthesis, and all of them can be affected in plants under DS. While under DS, plant roots encounter difficulty in absorbing sufficient moisture from the surrounding soil, and the water potential in leaves decreases. The DS damage to the plant mesophyll cell membrane and stomatal structure causes a series of reductions in plants, such as the CO_2_ concentration between the intercellular space of leaves, the chlorophyll concentration in chloroplasts, the photosynthetic rate, and the photosynthetic capacity [8,82].

With the application of certain ES, plants under DS are able to maintain a normal photosynthesis through stabilized stomatal closure, an increased photosynthetic rate, and enhanced transpiration intensity [83,84]. Among these ESs, melatonin can effectively improve plant biomass accumulation and photosynthesis under DS and enhance plant stress resistance [85]. γ-PGA enables plants under DS to use light energy more effectively through promoting the chlorophyll accumulation leaves and improving the stomatal conductance and photosynthesis [86]. The application of 6-BA alone can increase the stomatal conductance of plants, enhance the utilization efficiency of CO_2_, and escalate the net photosynthetic rate, while the combined application of 6-BA with ABA can promote the transport of photosynthetic products from leaves to roots [87]. It has been proven that 1.5 mM exogenous silicon increases the photosynthetic rate of plants and enhances the transpiration rate in leaves to mitigate the adversary effects of drought on plant photochemical reactions [88]. Exogenous calcium can stabilize the structure and function of chloroplasts, mitochondria, and cell membranes in mesophyll cells; maintain a normal net photosynthetic rate and gas exchange in leaves; and reduce the degree of degradation of photosynthetic pigments [89]. Teng [90] found that spraying 60 μM ABA on rice leaves could promote the upregulation of the expression of *OsPsbD1*, *OsPsbD2*, *OsNCED2*, *OsNCED3*, *OsNCED4,* and *OsNCED5* and the transcription of those genes in rice to improve the drought tolerance through increasing the photosynthetic rate and stomatal conductance. A pretreatment of the 100 mg L^−1^ 5-aminolevulinic acid could induce the transcription of *psbA* and *psbD* genes, thereby affecting the transcription of D1 protein, effectively repairing the function of PSII in the photosynthetic system, and alleviating the negative effects of DS on plant photosynthesis [91]. When adjusting their endogenous MDA and Pro concentrations in response to water shortage, plant roots under DS also change the amount and composition of their exudates accordingly, which indirectly affects the formation and activity of root microbial communities [92]. Fonseca [93] found that Bacillus subtilis could improve plant photosynthesis through affecting the synthesis of extracellular polymers and reducing the concentration of MDA and proline in sugarcane plants to alleviate DS damages. All ESs confirmed to improve plant photosynthesis are shown in Table 5.

### 3.4. Improvement of Plant Nutritional Status

Plant-required nutrients mainly include carbohydrates, lipids, proteins, and minerals. A sufficient supply of nutrients helps plants cope with drought through mediating photosynthesis, respiration, and protein synthesis. Being a carrier, water dissolves various nutrients and moves them around via vascular bundles in plants. Under DS, water absorption by plant roots is limited; the transpiration rate in plant leaves is reduced; the sap flow is decelerated; the nutrient influx via roots is reduced; and the mineral transport from roots to stems, leaves and reproductive organs is reduced [24]. Some ESs have been proven to improve plant drought tolerance through improving plant nutritional status either as required nutrients directly absorbed by plant roots or leaves or as promoting agents that regulate root biology and cell metabolism and improve the root absorption capacity for nutrients [98]. The exchange of plant exudates, minerals, nutrients, and so forth between plant roots and soil usually occurs in the rhizosphere, where abundant adapted microbes also grow and multiply. All these microbial activities are greatly affected and determined by root exudates that reshape the texture, components, and characteristics of the rhizosphere, thereby affecting the ability of plants to absorb nutrients and adapt to the environment [89]. Root exudates can regulate rhizosphere pH, ion concentration, and chemical properties of the solution, thereby affecting the availability of nutrients in the soil. In addition, the organic matter in root exudates can be used as a nutrient source for microorganisms to promote the growth and activity of beneficial microorganisms, thereby improving the rhizosphere micro-ecological environment and improving the health and growth of plants [99].

Soil drench of 1.5 mM silicon fertilizer (K_2_SiO_3_) before sowing significantly improved the root traits and functions of rice seedlings under DS and improved rice’s tolerance against drought [88]. Hosseini [100] found that supplying 540 g ha^−1^ calcium in the field could increase the content of Mg and Si in leaves and raise the concentration of putrescine and γ-aminobutyric acid (GABA) in leaves by positively regulating the polyamine pathway to achieve higher drought tolerance. Potassium fertilizer (K_2_SO_4_) at a 2.5 mM concentration proved to increase the content of K and other trace elements such as Fe, Zn, Cu, and Al in leaf tissues, as well as to improve drought resistance by extending the root longevity [101]. Spraying 2 mM GABA on the leaves under DS was able to increase the content of N, P, K, Ca, Fe, and Zn in the water-deficient leaves, thereby improving the nutrient acquisition and drought tolerance [61]. A non-reducing sugar, trehalose, at a 10 mM concentration was used in the foliar application to compensate for a shortage of total soluble sugars and promote the absorption of Ca^2+^ and K^+^ in shoots and roots under DS [102]. In addition, the abundance and activity of some rhizosphere microorganisms are closely related to and affect the nutrient intake capacity of roots; for example, inoculation of B. subtilis could increase the concentration of N, P, Mg, and S; generate more chlorophylls; escalate the photosynthetic rate; improve the water use efficiency; mitigate DS impact; accumulate more biomass; and enhance drought tolerance [93]. Under the circumstance that the soil pH, C/N ratio, and salt content have an impact on the composition of the soil microbial community [103,104], plant growth and tolerance against DS can be promoted through adding proper and adequate nutrients or ESs in soil to encourage changes in the microbial community and the activities against potential root impairments [105]. An arbuscular mycorrhiza inoculation has proven to increase the stomatal conductance; promote root development and growth under a water deficit; accelerate the absorption of nutrients such as nitrogen, phosphorus and potassium; and increase the production and yield of sunflower oil [106]. Liu et al. found that the microbial biomass was significantly reduced and the composition of microbial community greatly changed in dry soil, which was able to be altered through an application of exogenous phosphorus to increase the available phosphorus content in dry soil and alleviate damage to soybean under DS [107].

### 3.5. Phytohormone Regulation

The water condition in plants affects the level of endogenous hormones, which are essential and sensitive in receiving stress signals and initiating a chain of responses against DS. Among many phytohormones, it has been proven that ABA plays a critical role in sensing DS signals and converting them to chemical messages [108] and triggers a series of structural, biochemical, and physiological responses in plants. Burgess [109] found that when roots were under water shortage, ABA was the first phytohormone to initiate the stomatal response against DS through changing their cellular turgor pressure, triggering ABA accumulation near vascular bundles, transporting ABA to leaves, and adjusting stomatal functions, indicating that ABA is a signal carrier to transport cellular turgor pressure in roots to the forage [110,111]. Also, ABA has been found to induce oxidative responses and the production of antioxidants in regulation of leaf senescence through metabolic adjustments, and to modulate the CO_2_ input through stomatal conductance. Moreover, aquaporin seems to be involved in controlling the water absorption of roots under DS, while ABA has been proven to promote the expression of genes coding for aquaporin [112]. Under severe DS, the increased concentration of endogenous hormones activates various biochemical pathways for tolerance against intensified drought through promoting the ABA-mediated biosynthesis of osmotic substances such as proline. It is the consensus that the application of exogenous phytohormone such as ABA, IAA, or other exogenous substances (Si, PPi) before DS causes irreversible impairments to plants, which can be a life-saving strategy to protect plants under drought.

Other than ABA application, a foliar spray of 1 μM exogenous BR can significantly increase the endogenous ABA concentration in plants and regulate stomatal conductance and its upstream movement [113]. Cui [80] found that exogenous addition of lanthanum chloride could sustain and prolong the enzymatic activity of IAA and increase the levels of endogenous hormones such as IAA and GA3 in leaves. Sedaghat [114] used the synthetic strigolactone (SLS) analogue GR24 to drench wheat seedling roots and concluded that SLS treatments significantly increased the chlorophyll content and photosynthetic efficiency in seedlings under DS through enhancing the SLS signal transduction and ABA accumulation for strengthened drought tolerance. Xing [115] showed that a foliar spraying of 40 mg L^−1^ α-naphthylacetic acid could improve the dry matter quality of soybean shoots, increase their root-to-shoot ratios, promote the transport of sucrose from leaves to roots, and prevent the accumulation of soluble sugar caused by DS. Plant hormones are compounds synthesized by plants themselves, which play an important regulatory role in plant growth and development. However, in some cases, microorganisms that interact with plants can also produce plant hormones. Rhizobium sp. has been proven to produce C_2_H_4_-1-aminocyclopropane-1-carboxylate (ACC) deaminase that decomposes and absorbs ACC, which minimizes the ethylene production in roots, while it also stimulates the production of microbial extracellular polysaccharide (EPS) to improve the plant survival rate and induces the production of plant hormones such as IAA to promote root growth [116]. There are 11 ESs that have been confirmed to be useful in alleviating DS so far (Table 6).

## 4. Summary and Outlook

**Figure 3:**
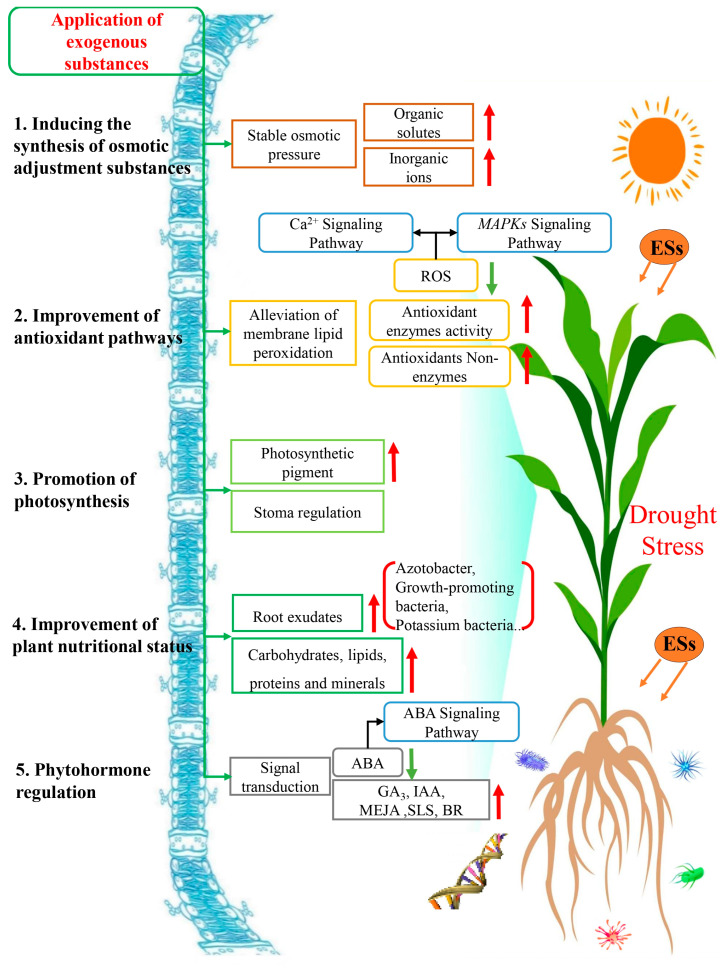
Mechanisms of exogenous substances involved in enhancing plant drought stress tolerance. Red and green arrows indicate promotion/increase or inhibition/decrease, respectively.

DS can affect the entire life cycle of a plant from its seed germination to maturity, affecting the morphological structures and physiological metabolisms through a series of adjustments from sensing the drought signal of a turgor pressure change in root cells and transmitting these signals (plant hormone, calcium ion concentration and ROS) to regulating stomatal closure, limiting CO_2_ influx, and reducing photosynthesis to slow down the growth and development. When DS continues and is prolonged, plants begin synthesizing and accumulating protective proteins and metabolites, regulating phytohormones, adjusting osmotic pressure, and enhancing the stability of cell membranes to protect cells from dehydration and avoid degradation or abnormal folding of albumin. Under a worst DS scenario, plants may activate a series of protective mechanisms such as regulating ion balance, improving antioxidant capacity, and accumulating expression of stress-related genes to avoid damage caused by drought stress. However, plant drought tolerance in response to DS, as elaborated above, is rather limited and insufficient when the drought situation is intensified. Therefore, it is a rational and practical strategy to supplement ESs or some beneficial microorganisms that plants under DS need to boost their drought tolerance against DS. Numerous studies have proven that applying ESs can effectively alleviate the damage caused by DS, and they have alleviated DS in five categories, as described and discussed above, but these modes of action are interacted, intertwined, and convoluted (Figure 3). In responding to DS, these five mechanisms described in plants do not take place in a strict and sequential order, but function as a whole in a dynamic process temporally and spatially; they are flexible, adjustable, and objective to the severity and duration of DS [62]. For example, in the early stages of drought, plants initially induce the synthesis of osmotic regulatory substances, such as proline, betaine, etc., to increase intracellular osmotic pressure, reduce water loss, and help cells maintain turgor pressure [18]. At this time, some ESs can be used to enhance plant DS tolerance through maintaining the stability of cell membranes by inducing more osmoregulatory substances, increasing solute cells, reducing cell membrane permeability, and keeping the osmotic pressure under check. When DS is prolonged and intensified, the antioxidant pathways are promoted to further accumulate more osmoregulatory substances, such as antioxidant enzymes (SOD, POD, CAT, etc.), to scavenge ROS, reduce oxidative damage [40], and improve the photosynthesis efficiency [82], which can be accomplished by supplementation of K^+^ and other minerals to scavenge free radicals, reduce oxidative damage, enhance the sugar metabolism pathway, increase cell osmotic pressure, and maintain the functions of the stomata.

Plants exhibit various degrees of tolerance against DS at different growing stages, such as germination, seedling, growing, blossoming, and fruiting. DS due to a water shortage can reduce the seed germination rate and impair its emergence process. When DS is mild, seedlings seem more vulnerable and sensitive to the water deficit, but this can be an ideal period to prime for drought tolerance. Thus, to date, most studies on the alleviation DS through using ESs have been carried out on seedlings. However, severe and persistent drought can ultimately cause severe and irreversible damage to the roots, leaves, flowers and fruit, resulting in stomatal closures, leaf wilting, weakened photosynthesis, decreased transpiration, and eventually death. Therefore, the use of ESs to alleviate the impact due to DS, fortify plant resistance/tolerance against DS, and prime seedlings for prolonged drought at all growing stages should be further focused on and attempted in our research.

The method, timing, and targeted plant part of ES application can be essential for ES’s effectiveness in alleviating plant DS. Foliar spray, seed soaking, and root drench have been commonly used to study ES’s effect on mitigating the DS impact. To achieve an ultimate efficacy of ESs on plant DS alleviation, the method and concentration of each ES are carefully chosen based on the characteristics of various crops. For the foliar application, the thick leaf stratum corneum and wax layer that some drought tolerant plants have to better adapt to the dry environment can block the absorption of ESs from entering the epidermis and mesophyll tissue inside the leaf. Fernández [121] found that the absorption of ESs on the lower surface of leaves was faster and more effective, indicating that more ES solutions should be applied onto the lower leaf surface. For the root drench application, ESs should be considered prior to the onset of drought when irrigation water is still available to prime roots for a water deficit in soil and enhance plant tolerance against DS. Moreover, related studies have shown that a combined application of two ESs can synergize the effects of both ES and complement their different mode of actions for a better efficacy, such as a joint application of ABA and melatonin [122], MeJA and SA [123], and Si plus H_2_S [34], which have proven to be more effective in enhancing plant drought tolerance. So far, less effort has been focused on understanding the ES’s efficacy through adjustment of its application method, timing, duration, and targeted plant parts, etc., in a field setting. A more thorough understanding of all the aspects involved in the mechanisms that underlie the ES effect on plant DS mitigation will shed light on how ES works regarding plant tolerance against DS.

The research on ESs for past two decades has mainly focused on plant-associated hormones, growth regulators, and ions (Table 2). When DS being sensed, plants respond through their internal regulatory substances, such as ABA, which first reduces the transpiration by promoting stomatal closure to minimize water loss, then, as a signal, induces the synthesis of antioxidant enzymes to remove excessive ROS and prevent oxidative damage. Finally, as a messenger, it induces the expression of a series of genes encoding for proteins and enzymes involved in water transport, osmoregulation, and antioxidant defense [108,109,110,111,112]. Therefore, the application of exogenous ABA can initiate the conduction of cytoplasmic membranes in leaf cells, induce uneven closure of foliar stomata, reduce water transpiration and loss, and balance endogenous hormones required to improve plant water retention capacity and drought tolerance [90]. Moreover, H_2_O_2_ in plants can act as a secondary messenger participating in regulating plant response to DS to activate a range of signal transduction pathways, including Ca^2+^ signaling and MAPKs signaling, etc. The application of exogenous H_2_O_2_ to plants can promote the accumulation of osmo-modulators, such as proline and soluble sugars, by activating signaling pathways, thereby helping plants to maintain cell osmotic pressure and water balance [35].

Obviously, research on deciphering the underlying mechanisms involved in plant drought tolerance against DS has progressed greatly, but more studies should focus on the following in the future: (1) understanding if the foliar application of ES is more effective than root drench through revealing how an ES penetrates through leaf cuticles and waxes, moves to roots, and works in root meristems; (2) clarifying how GABA, H_2_O_2_, and other signaling molecules activate gene expression and signal transduction; (3) seeking a potential use of more than one ES for a synergetic effect on the basis of the individual mechanism(s) involved in mitigating plant DS, or a universal ES for many crops; (4) improving the efficacy of ESs through optimization of the application method, timing, concentration, and target plant parts, etc.; (5) exploring the possible use of genomics, proteomics, transcriptomics, and other novel techniques to better explain the molecular basis of plant drought tolerance and improve the water use efficiency of plants under DS; and (6) combining newly advanced technologies such as microorganisms, hydrogels, nanoparticles, and biological metabolic engineering technology to use ESs for plant DS alleviation. We strongly believe that, under the circumstances of continuous climate change, global warming, and more erratic onsets of drought, researchers are tasked more than ever with facing the challenges brought forth by these adversary environmental conditions. Using ESs to alleviate crop DS can be one of many strategies to mitigate the potential crop loss due to intensified drought and secure the worldwide food supply.

## Figures and Tables

**Table 1:** List of exogenous substances used for alleviation of plant drought stress.

Polyamine		
Spermidine (Spd)	Putrescine (Put)	
Polysaccharide		
Chitosan (CTS)	Trehalose	
Polyphenol		
Anthocyanin (AC)		
Polypeptide		
Glutathione (GSH)		
Polyol		
Sorbitol (ST)	Inositol	
Amino Acid		
Glycine Betaine (GB)	5-Aminolevulinic acid (5-ALA)	Arginine (Arg)
*γ*-Aminobutyric acid (GABA)	*γ*-polyglutamic acid (*γ*-PGA)	*β*-aminobutyric acid (BABA)
Proline (Pro)		
Organic Acid		
Humic acid (HA)	Fulvic acid (FA)	Salicylic acid (SA)
*α*-Lipoic Acid (ALA)	Cinnamic acid (CA)	Acetic acid (HAc)
Citric acid	Ferulic acid (FA)	
Phytohormone		
S-excitation (S-ABA)	Ethephon (ETH)	Gibberellins (GA_3_)
Abscisic acid (ABA)	Melatonin (MT)	Brassinolide (BR)
Methyl jasmonate (MeJA)	Cytokinin (CTK)	Strigolactones (SLS)
6-Benzylaminoadenine (6-BA)		
Plant-growth Regulator		
Coronatine (COR)	Paclobutrazol (PBZ)	1-methylcyclopropene (1-MCP)
*α*-naphthaleneacetic acid (NAA)	2-(3,4-Dichlorophenoxy) triethylamine (DCPTA)	Choline chloride (Cc)
Signaling Molecule		
Nitric oxide (NO)	Hydrogen sulfide (H_2_S)	Methylglyoxal (MG)
Hydrogen peroxide (H_2_O_2_)	Carbon monoxide (CO)	
Element		
Calcium (Ca)	Potassium Kalium (K)	Selenium (Se)
Silicon (Si)	Boron (B)	Phosphorus (P)
Compound		
Lanthanum nitrateon	lanthanum chloride	Sodium chloride (NaCl)
Microorganism		
Arbuscular mycorrhizal	Rhizobium	NBRIRA
NBRISN13	Bacillus subtilis	
Other		
AgNPs	Paraquat (PQ)	Seaweed extract (SWE)

**Table 2:** List of the most frequently mentioned and studied exogenous substances and their underlying mechanisms involved in alleviation of plant drought stress for last 24 years (Web of Science™).

Exogenous Substance	No. of Papers (Topic Search)	No. of Papers (Refined by MeSH)	Mechanisms Involved
Abscisic acid (ABA)	1547	420	2, 3, 5
Hydrogen peroxide (H_2_O_2_)	855	147	1, 2
Glutathione (GSH)	379	44	2
Melatonin (MT)	346	124	2, 3
Nitric oxide (NO)	332	62	2
Potassium kalium (K)	279	16	1, 3, 4
Calcium (Ca)	273	28	3, 4
Glycine betaine (GB)	185	16	1
Spermidine (Spd)	183	28	1, 5
Methyl jasmonate (MeJA)	160	29	1, 5

Note: 1. Induced synthesis of osmotic regulators; 2. improvement of antioxidant pathways; 3. promotion of photosynthesis; 4. improvement of plant nutritional status; 5. phytohormone regulation.

**Table 3:** Application methods and the optimal concentration of exogenous substances used for inducting the synthesis of osmotic regulators.

Exogenous Substance	Optimal Concentration	Application Method	Crop	Mechanism of Exogenous Substances	Reference
Putrescine	100 μM	Hydroponics	Wheat (*Triticum aestivum* L.)	An effective reduction in the osmotic stress and increase in the photosynthetic capacity and proline concentration of crops under DS.	[26]
Chitosan	100 mg L^−1^	Foliar spraying	Wheat (*Triticum aestivum* L.)	Maintains the integrity and stability of the cell membranes of seedlings and increases the proline concentration and soluble protein content through foliar spraying at the early stage of drought to increase the soluble sugar content in leaves for a better osmotic adjustment.	[22]
Anthocyanin	4 mgL^−1^	Foliar spraying	Tobacco (*Nicotiana tabacum* L.)	Used to promote the accumulation of sucrose and amino acids, especially proline, and alleviate osmotic impairments.	[27]
Glycine Betaine	100 mM	Foliar spraying	Wheat (*Triticum aestivum* L.)	Enhances the osmotic adjustment, increases the photosynthetic rate, upregulates the STI gene of wheat genotypes, and accumulates more proline and endogenous betaine through reducing the sucrose content in leaves.	[28]
*γ*-polyglutamic acid	20 mgL^−1^	Hydroponics	Rice (*Oryza sativa* L.)	An increase in the proline concentration and stabilization of plant osmotic pressure.	[20]
Proline	30 mM	Foliar spraying	Rice (*Oryza sativa* L.)	Increases the activity of total soluble protein, proline, and glycine betaine in leaves, promotes the K^+^ absorption efficiency, and produces more osmotic protective agents.	[29]
Humic acid	200 mg L^−1^	Seed soaking	Millet (*Setaria italica* (L.) Beauv.)	Promotes the accumulation of soluble protein and free proline and improves the osmotic adjustment ability.	[30]
cinnamic acid	50 μM	Drench	Cucumbers (*Cucumis sativus* L.)	Increases the contents of ascorbic acid, proline, soluble sugar, vanillic acid (VA), and CA in leaves and enhances the osmotic adjustment ability.	[31]
Brassinolide	1 μM	Foliar spraying	wheat (*Triticum aestivum* L.)	A positive effect on maintaining photosynthetic capacity and initiating osmotic protection and other hormone induction.	[32]
Methyl jasmonate	0.5 mM	Foliar spraying	Wheat (*Triticum aestivum* L.)	Used for promoting the accumulation of total soluble sugars, polysaccharides and carbohydrates, enhancing the accumulation of osmotic protective agents, initiating stomatal closures, escalating the water use efficiency, and inducing the transport of assimilates to increase yield.	[33]
Hydrogen sulfide	0.3 mM	Foliar spraying	Cucumbers (*Cucumis sativus* L.)	Promotes the total soluble sugar, protein, and proline content, improves the osmotic adjustment ability, and alleviates oxidative damages.	[34]
Hydrogen peroxide	1.5 mM	Foliar spraying	Cucumbers (*Cucumis sativus* L.)	Promotes accumulating soluble sugar and proline, improves osmotic adjustments, and enhances antioxidant defenses and photosynthesis.	[35]
Potassium Kalium	80 mgkg^−1^	Fertilization	Rape (*Brassica napus* L.)	Increases the root length and root density, balances the root-to-shoot ratio to increase root water uptake potential, stimulates the root secretion of organic acids, and promotes nutrient acquisition and utilization.	[36]
Selenium	40 mg L^−1^	Foliar spraying	Wheat (*Triticum aestivum* L.)	It affects the accumulation of osmotic adjustment substances, reduces osmotic potential, and promotes the accumulation of soluble sugar and free amino acids.	[37]
Silicon	2 mM	Fertilization	Wheat (*Triticum aestivum* L.)	Enhances osmotic adjustment ability; increases aquaporin activity, root water conductivity, and leaf water content; and promotes the nutrient renewal and photosynthetic rate.	[38]
Sodium chloride	100 mM	Drench	Grass leaved orache (*Atriplex patens*)	Increases the content of glycine betaine and soluble sugar in leaves, accumulates Na^+^ to enhance water absorption, and reduces the retention of Na^+^ in photosynthetic organs, thereby protecting cell membrane and structure.	[21]

**Table 4:** Application methods and the optimal concentration of exogenous substances used to induce antioxidant enzymes.

Exogenous Substance	Optimal Concentration	Application Method	Crop	Mechanism of Exogenous Substances	Reference
Spermidine	1 mM	Drench	Wheat (*Triticum aestivum* L.)	Used to promote the activities of SOD, POD and CAT in plant grains and reduce the MDA content.	[55]
Putrescine	100 μM	Hydroponics	Wheat (*Triticum aestivum* L.)	Used to alleviate oxidative damage, improve the antioxidant capacity, and enhance the GST and POD activity.	[26]
Chitosan	100 mg L^−1^	Foliar spraying	Potato (*Solanum tuberosum*)	Used to increase the activity of SOD and POD in leaves, remove the active oxygen, and stabilize cell membranes.	[22]
Trehalose	30 mM	Foliar spraying	Maize (*Zea mays* L.)	Used to reduce malondialdehyde and SOD activity, and increase the POD and CAT activity.	[56]
Glutathione	0.2 mM	Hydroponics	Cotton (*Gossypium* spp.)	Used to inhibit the accumulation of ROS in plant cells; upregulate genes coding for enzymatic and non-enzymatic antioxidants such as CAT, ascorbate peroxidase (APX), peroxidase (POX), reduce ascorbic acid (AsA), glutathione peroxidase (GSH), etc.; mitigate the severity of ROS-induced oxidative damage; and decrease H_2_O_2_ and malondialdehyde (MDA) accumulation.	[57]
Sorbitol	10 mM	Foliar spraying	Maize (*Zea mays* L.)	Used to increase the activity of SOD, POD, CAT, and ASA, GSH and enhance the antioxidant capacity.	[58]
Inositol	15 μM	Foliar spraying	Capsicum (*Capsicum annuum* L.)	Used to reduce the MDA content and enhance scavenging ROS by increasing POD and GR activities.	[59]
5-Aminolevulinic acid	75 mgL^−1^	Foliar spraying	Sunflower (*Helianthus annuus* L.)	Used to increase chlorophylls and the activity of antioxidant enzymes (APX, SOD and CAT).	[45]
Arginine	0.1 mM	Hydroponics	Wheat (*Triticum aestivum* L.)	Used to increase the activity of CAT, GPX, GST, and other antioxidant enzymes, and to increase the endogenous NO content for the purpose of regulating the antioxidant system and reducing ROS production.	[60]
*γ*-Aminobutyric acid	2 mM	Foliar spraying	Bean (*Phaseolus vulgaris* L.)	Used to increase the activity of SOD, CAT, POX, and APX.	[61]
Proline	30 mM	Foliar spraying	Rice (*Oryza sativa* L.)	Used to promote the activity of SOD, POD, and CAT and improve the antioxidant capacity.	[29]
Humic acid	200 mg L^−1^	Seed soaking	Millet (*Setaria italica* (L.) Beauv.)	Used to reduce H_2_O_2_ and increase the activity of SOD, POD, and CAT.	[30]
Fulvic acid	1.5 mgL^−1^	Foliar spraying	Maize (*Zea mays* L.)	Used to increase the SOD, POD, and CAT activity and proline level and to maintain the chlorophyll content and gas exchange rate.	[62]
*α*-Lipoic Acid	0.02 mM	Seed soaking	Wheat (*Triticum aestivum* L.)	Used to enhance the activity of SOD, APX, CAT, and POX and remove ROS.	[63]
Cinnamic acid	50 μM	Drench	Cucumbers (*Cucumis sativus* L.)	Used to enhance the activity of GPX, glutathione peroxidase (GSH-Px), DHAR, and GR and reduce the lipid peroxidation.	[31]
Citric acid	50 mM	Foliar spraying	Tobacco (*Nicotiana tabacum* L.)	Used to enhance the activity of POD and CAT and reduce the ROS accumulation.	[64]
Ferulic acid	0.5 mM	Drench	Cucumbers (*Cucumis sativus* L.)	Used to inhibit the ROS production; reduce the MDA content; induce the activity of SOD, CAT, GPX, GSH, and APX in leaves; and increase the proline and soluble sugar content.	[65]
S-excitation	1000-fold	Seed soaking	Oat (*Avena sativa* L.)	Used to reduce the MDA content and increase the antioxidase activity.	[66]
Ethephon	1.0 mM	Foliar spraying	Maize (*Zea mays* L.)	Used to reduce MDA and hydrogen peroxide and to increase proline and the activities of SOD, POD, and CAT, thereby reducing oxidative damage and maintaining membrane integrity and stability.	[67]
Abscisic acid	60 μM	Foliar spraying	Kiwi fruit (*Actinidia*)	Used to keep cell membranes undamaged and promote the activity of the POD, CAT, SOD, APX, and GR antioxidant enzymes.	[68]
Melatonin	0.1 mM	Foliar spraying	Tomato (*Solanum lycopersicum*)	Used to decrease the MDA content and increase the activity of POD, SOD, CAT, APX, and GR, as well as the content of ASA.	[69]
Brassinolide	1 μM	Foliar spraying	Wheat (*Triticum aestivum* L.)	Used to reduce the MDA content and alleviate oxidative stress.	[32]
Paclobutrazol	2 mM	Hydroponics	Stevia (*Stevia rebaudiana*)	Used to reduce membrane damage, prevent the leakage of electrolytes, and decrease the MDA level.	[70]
1-Methylcyclopropene	2.4 g L^−1^	Gas	Cotton (*Gossypium* spp.)	Used to maintain the integrity of cell membranes through increasing the activity of antioxidant enzymes such as POD.	[71]
2-(3,4-Dichlorophenoxy) triethylamine	15 mM	Hydroponics	Maize (*Zea mays* L.)	The activities of POD and CAT are enhanced, and the accumulation of reactive oxygen species is inhibited.	[72]
Nitric oxide	100 μM	Foliar spraying	Soybean (*Glycine max* (Linn.) Merr)	Used to improve the activity of the SOD, CAT, APX, and POX antioxidant enzymes and alleviate oxidative damage.	[73]
Hydrogen sulfide	0.3 mM	Foliar spraying	Wheat (*Triticum aestivum* L.)	Used to reduce the content of H_2_O_2_ and MDA and improve the oxidative stress tolerance through increasing the activity of antioxidant enzymes.	[34]
Methylglyoxal	15–25 mM	Foliar spraying	Maize (*Zea mays* L.)	Used to inhibit the accumulation of endogenous MG and activate the glyoxalase system in leaves.	[74]
Strigolactones	20 μM	Foliar spraying	Maize (*Zea mays* L.)	Used to enhance the activity of antioxidant enzymes such as POD, SOD, CAT, and APX.	[75]
Hydrogen peroxide	1.5 mM	Foliar spraying	Cucumbers (*Cucumis sativus* L.)	Used to increase the activity of SOD and POD and improve the ability of leaves to scavenge ROS.	[35]
Carbon monoxide	0.1 μM	Hydroponics	Rice (*Oryza sativa* L.)	Used to promote the activity of SOD, CAT, and POD in leaves and enhance the antioxidant capacity.	[76]
Selenium	40 mg L^−1^	Foliar spraying	Wheat (*Triticum aestivum* L.)	Used to promote the activity of antioxidant enzymes such as CAT, POX, and APX.	[37]
Silicon	2 mM	Fertilization	Wheat (*Triticum aestivum* L.)	Used to increase the activity of SOD, CAT, APX and POD and alleviate the damage of oxide film.	[38]
Boron	50 μM	Hydroponics	Tomato (*Solanum lycopersicum*)	Used to reduce the MDA content and increase the APX and glutathione reductase (GR) activity.	[53]
Lanthanum nitrateon	10 mM	Foliar spraying	Tomato (*Solanum lycopersicum*)	Used to increase the activity of all enzymes involved in the ASC-GSH cycle.	[77]
Lanthanum chloride	400 mM	Seed soaking	Maize (*Zea mays* L.)	Used to enhance the activity of POD, CAT, and SOD.	[78]
AgNPs	0.1 μM	Foliar spraying	Egg plant (*Solanum melongena* L.)	Used to increase the content of H_2_O_2_ and MDA and to promote the activity of SOD and CAT antioxidant enzymes.	[79]
Paraquat	10 mM	Drench	Cucumbers (*Cucumis sativus* L.)	Used to increase the activity of SOD, CAT, GPX, APX, DHAR, MDHAR, GR, GSH, and AsA.	[80]
Seaweed extract	0.5 L/ha	Fertilization	Cane (*Saccharumofficinarum* L.)	Increases the activity of SOD, POD, CAT, and PPO, and promotes the balance of ROS.	[81]

**Table 5:** Application methods and the optimal concentrations of exogenous substances used to promote photosynthesis.

Exogenous Substance	Optimal Concentration	Application Method	Crop	Mechanism of Exogenous Substances	Reference
5-Aminolevulinic acid	10 mgL^−1^	Seed soaking	Alfalfa (*Medicago sativa* L.)	Used to increase the chlorophyll content and photosynthetic rate of leaves, decrease the stomatal density of cotyledons, and increase the stomatal width.	[94]
*γ*-polyglutamic acid	50 mgL^−1^	Fertilization	Maize (*Zea mays* L.)	Used to promote the accumulation of chlorophyll and light and parameters and improve the net photosynthetic rate and stomatal conductance.	[86]
*β*-aminobutyric acid	75 mM	Foliar spraying	Sunflower (*Helianthus annuus* L.)	Used to promote the green retention by increasing SPAD-chlorophyll content.	[95]
Humic acid	200 mg L^−1^	Seed soaking	Millet (*Setaria italica* (L.) Beauv.)	Used to repair damages to chlorophylls, prevent chlorophyll degradation, increase the stomatal conductance, and increase the photosynthetic rate.	[30]
Abscisic acid	60 μM	Foliar spraying	Rice (*Oryza sativa* L.)	Used to improve photosynthesis through accumulating chlorophyll fluorescence and upregulating the expression of chloroplast genes.	[90]
Melatonin	0.1 mM	Foliar spraying	Tomato (*Solanum lycopersicum*)	Used to enhance the chlorophyll metabolism of leaves, promote the accumulation of chlorophyll, delay the decomposition process, and maintain a high level of photosynthetic efficiency.	[69]
6-Benzylaminoadenine	60 mgL^−1^	Foliar spraying	Sweetpotato (*Dioscorea esculenta* L.)	Used to increase the stomatal conductance, CO_2_ utilization, and net photosynthetic rate.	[87]
Choline chloride	2.1 mM	Foliar spraying	Rehmannia glutinosa (*Rehmannia*)	Used to maintain a high level of leaf water content, delay leaf water loss, and promote the recovery of photosynthesis after rehydration.	[96]
Hydrogen peroxid	1.5 mM	Foliar spraying	Cucumbers (*Cucumis sativus* L.)	Used to increase the chlorophyll content and leaf water content and improve the photosynthetic capacity.	[35]
Calcium	10 mM	Foliar spraying	Tobacco (*Nicotiana tabacum* L.)	Used to stabilize the structure and function of chloroplast, mitochondria and inner membranes in mesophyll cells, maintain the normal net photosynthetic rate and gas exchange in leaves, and minimize the degradation of photosynthetic pigments.	[89]
Potassium Kalium	2.5 mM	Fertilization	Hibiscus (*Hibiscus syriacus* L.)	Used to promote the increase in K^+^ concentration in chloroplasts to maintain the photosynthetic activity.	[97]
Silicon	1.5 mM	Fertilization	Rice (*Oryza sativa* L.)	Used to improve the photosynthetic rate and mitigate impairments on photochemical reactions.	[88]

**Table 6:** Application methods and the optimal concentration of phytohormones used as exogenous substances to alleviate crop drought stress.

Exogenous Substance	Optimal Concentration	Application Method	Crop	Mechanism of Exogenous Substances	Reference
Spermidine	1 mM	Pouring	Wheat (*Triticum aestivum* L.)	Used to regulate the endogenous hormone level, promote the synthesis of cytokinin (CTK) and starch in grains, and reduce the synthesis of ethylene.	[55]
Acetic acid	8 mM	Pouring	Cotton (*Gossypium* spp.)	Used to activate more gene expression for higher levels of endogenous ABA and JA.	[117]
Gibberellins	50 mgL^−1^	Foliar spraying	Maize (*Zea mays* L.)	Used to increase the division of damaged cells, promote the enzyme activity, and balance the endogenous hormones through increasing the content of endogenous gibberellin.	[118]
Abscisic acid	60 μM	Foliar spraying	Rice (*Oryza sativa* L.)	Used to regulate stomatal opening and closure, reduce transpiration, and inhibit the synthesis of endogenous ABA to balance the changes of endogenous hormone levels.	[90]
Brassinolide	1 μM	Foliar spraying	Wheat (*Triticum aestivum* L.)	Used to maintain normal photosynthetic activity, produce more antioxidants, generate osmotic protection, and induce other hormones involved in DS alleviation.	[32]
Methyl jasmonate	0.5 mM	Foliar spraying	Wheat (*Triticum aestivum* L.)	Used to affect the synthesis of endogenous hormones in plants, promote the accumulation of total soluble sugars, polysaccharides, carbohydrates, and osmotic protective agents; enhance the stomatal closure; improve the water use efficiency; and induce the transport of assimilates to increase yield.	[33]
Cytokinin	100 μM	Foliar spraying	Creeping herbs (*Agrostis stolonifera* L.)	Used to repair damages due to membrane lipid peroxidation and promote the nitrogen metabolism.	[119]
Strigolactones	20 μM	Foliar spraying	Maize (*Zea mays* L.)	Used to increase the leaf water content and chlorophyll content and improve the activity of antioxidant enzymes.	[75]
Coronatine	10 μM	Foliar spraying	Wheat (*Triticum aestivum* L.)	Used to boost the JA or jasmonate signaling pathway and ABA accumulation and to regulate the balance of endogenous hormones.	[120]
*α*-naphthaleneacetic acid	40 mgL^−1^	Foliar spraying	Soybean (*Glycine max* (Linn.) Merr)	Used to promote the movement of soluble sugar from leaves to roots, promote the distribution of photosynthetic assimilates to sucrose, inhibit the conversion of sucrose to starch and hexose, increase the photosynthetic rate, and reduce the inhibition of photosynthesis.	[115]
Hydrogen sulfide	0.3 mM	Foliar spraying	Wheat (*Triticum aestivum* L.)	Used to increase the endogenous SA content, regulate the endogenous ABA content, and maintain the hormone balance.	[34]
lanthanum chloride	400 mM	Seed soaking	Maize (*Zea mays* L.)	Used to improve the photosynthetic rate and antioxidant enzymes and to change the levels of endogenous hormones such as auxin and gibberellin during the reproductive period.	[78]

## Data Availability

Not applicable.
